# Renal Ischemia/Reperfusion Mitigation via Geraniol: The Role of Nrf-2/HO-1/NQO-1 and TLR2,4/MYD88/NFκB Pathway

**DOI:** 10.3390/antiox11081568

**Published:** 2022-08-13

**Authors:** Maged E. Mohamed, Mohammad A. Elmorsy, Nancy S. Younis

**Affiliations:** 1 Al Bilad Bank Scholarly Chair for Food Security, The Deanship of Scientific Research, The Vice Presidency for Graduate Studies and Scientific Research, Al-Ahsa 31982, Saudi Arabia; 2 Department of Pharmaceutical Sciences, College of Clinical Pharmacy, King Faisal University, Al-Ahsa 31982, Saudi Arabia; 3 Department of Pharmaceutical Organic Chemistry, Faculty of Pharmacy, Mansoura University, Mansoura 35516, Egypt; 4 Department of Pharmaceutical Chemistry, Faculty of Pharmacy, Delta University for Science and Technology, Gamesa 35712, Egypt

**Keywords:** anti-inflammatory, antioxidant, essential oil, geraniol, renal ischemia/reperfusion

## Abstract

Background: Renal ischemia/reperfusion injury is a clinically recurrent event during kidney transplantation. Geraniol is a natural monoterpene essential oil component. This study aimed to inspect geraniol’s reno-protective actions against renal I/R injury with further analysis of embedded mechanisms of action through scrutinizing the Nrf-2/HO-1/NQO-1 and TLR2,4/MYD88/NFκB signaling pathways. Methods: Wistar male rats were randomized into five groups: Sham, Sham + geraniol, Renal I/R, and two Renal I/R + geraniol groups representing two doses of geraniol (100 and 200 mg/kg) for 14 days before the renal I/R. Renal I/R was surgically induced by occluding both left and right renal pedicles for 45 min, followed by reperfusion for 24 h. A docking study was performed to anticipate the expected affinity of geraniol towards three protein targets: hTLR4/MD2, hTLR2, and hNrf2/Keap1. Results: Renal I/R rats experienced severely compromised renal functions, histological alteration, oxidative stress status, escalated Nrf-2/HO-1/NQO-1, and amplified TLR2,4/MYD88/NFκB. Geraniol administration ameliorated renal function, alleviated histological changes, and enhanced Nrf-2/HO-1/NQO-1 with a subsequent intensification of antioxidant enzyme activities. Geraniol declined TLR2,4/MYD88/NFκB with subsequent TNF-α, IFN-γ, MCP-1 drop, Bax, caspase-3, and caspase-9 reduction IL-10 and Bcl-2 augmentation. Geraniol exhibited good fitting in the binding sites of the three in silico examined targets. Conclusions: Geraniol might protect against renal I/R via the inhibition of the TLR2,4/MYD88/NFκB pathway, mediating anti-inflammation and activation of the Nrf2 pathway, intervening in antioxidative activities.

## 1. Introduction

Renal ischemia/reperfusion (I/R) injury is a clinically frequent incident during kidney transplantation. I/R injury often initiates non-specific inflammatory responses that can result in the loss of kidney graft viability during renal transplantation [1]. I/R-induced renal injury with accompanied renal dysfunction may cause renal failure and renal-cell death [2]. During the ischemic phase of renal I/R, the inadequate oxygen supply causes mitochondrial dysfunction [3]. Reperfusion of the ischemic kidney further worsens the state of oxidation and inflammation, resulting in necrosis or apoptosis by damaging cellular DNA, protein, and cellular integrity. The underlying pathophysiological mechanisms involved in renal I/R induced-injury include releasing reactive oxygen species (ROS), generating pro-inflammatory mediators, calcium overload, and activating apoptotic genes [4]. The ROS detrimental outcome on renal tissues has been considered fundamental in renal injury pathophysiological processes [5]. Oxygen radicals manufactured after ischemia/reperfusion can cause lipid peroxidation and destroy cell and organelle membranes, disturbing renal tissue structure and function. Nuclear factor-erythroid factor 2-related factor 2 (Nrf2) is a redox-sensitive transcriptional factor that sustains cellular defense via its role as an antioxidant and an anti-inflammatory and via its cytoprotective actions [6]. During oxidative stress, Nrf2 separates from its cytoplasmic inhibitor protein (KEAP1) to translocate into the nucleus, initiating numerous antioxidant enzymes, e.g., HO-1, NQO-1, glutathione peroxidase, superoxide dismutase, and catalase. Several studies described the Nrf2 pathway shielding role in different organ-ischemia models, such as the kidney [7], heart [8], brain [7], and liver [6].

Hypoxic and anoxic cell injuries arise within the renal tissue after ischemia, leading to vigorous inflammatory cytokines production. These cytokines initiate defensive physiological actions to separate and inhibit tissue damage or further exacerbate organ dysfunction via free radical production and inflammatory cell recruitment [4]. Danger signals released by dying cells alarm Toll-like receptors (TLR) that transmit the signal to transcription factors through adapter molecules and a chain of kinases, which encode the genes regulating inflammatory cells and mediators. 

**Figure 1:**
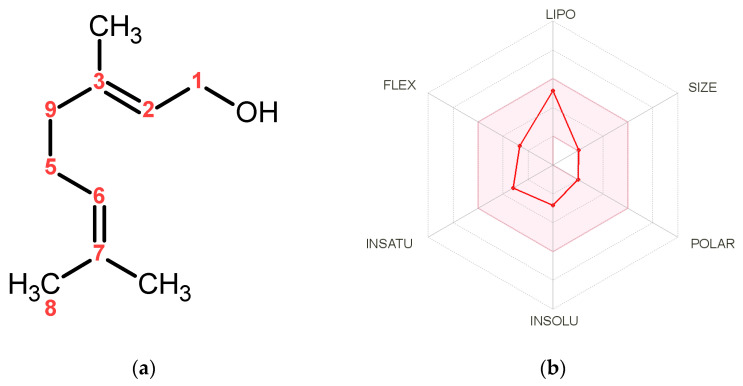
(**a**) Geraniol chemical structure. (**b**) Pharmacokinetics and bioavailability radar graph of geraniol. The data were calculated in silico using the SwissADME web tool [20]. The pink area in the radar graph represents the optimal range for each particular property for studied compounds (LIPO = lipophilicity as XLOGP3; SIZE = size as molecular weight; POLAR = polarity as TPSA (topological polar surface area); INSOLU = insolubility in water by log S scale; INSATU = in saturation as per fraction of carbons in the sp3 hybridization and FLEX = flexibility as per rotatable bonds).

Geraniol (Figure 1a), an acyclic monoterpene alcohol, is the main constituent of the essential oils of rose, lavender, lemon, and orange [9]. The pharmacological attributes of geraniol involve a broad scale of properties, including cytotoxic and antitumor [10], anti-inflammatory [11], immunomodulatory, antioxidant [6], hepatoprotective [6], nephroprotective [12], neuroprotective [13], and cardioprotective [14]. Several studies documented that geraniol controls’ various signaling pathways involved in the cell cycle, oxidative stress, inflammation, apoptosis, and autophagy. For instance, geraniol mitigates diabetic nephropathy via interference with the miRNA-21/PTEN/Akt/mTORC1 pathway in rats [12]. The compound ameliorates TNBS-induced colitis via its antioxidant, anti-inflammatory, and immunosuppressive potential, possibly by modulating the Wnt/GSK-3β/β-catenin, p38MAPK, NFκB, and PPARγ signaling pathways. [15]. Furthermore, geraniol activated the Nrf-2/HO-1 signaling pathway, mediating protection against oxidative stress-induced apoptosis in the hepatic ischemia-reperfusion injury [6].

It is recognized that inflammation and oxidative stress, altogether, are perhaps the most crucial pathophysiological processes involved in the propagation of renal I/R injury [16]; hence, it is imperative to control oxidative stress and inflammation during renal I/R. Therefore, effective measures to attenuate kidney I/R injury may improve patients’ postoperative survival. Several studies have shown that early controlling of the inflammatory response could be a promising way to avert renal I/R injury [17,18,19]. In this context, and recognizing that the potential nephroprotective effect of geraniol against renal ischemia-reperfusion has not been yet enlightened, despite the compound’s efficacy on different disorders, we, hereby, in this study, investigated the influence of geraniol on renal I/R injury and explored the possible underlying mechanisms, including Nrf-2/HO-1/NQO-1 and TLR2,4/MYD88/NFκB signaling pathways.

## 2. Materials and Methods

### 2.1. Absorption, Distribution, Metabolism, and Excretion Assessment of Geraniol

Geraniol was subjected to absorption, distribution, metabolism, and excretion (ADME) estimate using the SwissADME server applying the compound in SMILES format. SwissADME [20] is a software supported by SIB Swiss Institute of Bioinformatics that computes the physicochemical properties and predicts the pharmacokinetics and drug-likeness of various small molecules. 

### 2.2. Animals

Wistar male rats (age: 4–6 weeks; weight: 200–240 g) were purchased from Experimental Animal Research Centre, King Saud University, Riyadh, KSA. Animals were provided with standard laboratory food and water ad libitum. Animals were kept in ventilated cage system (12 h light/dark cycle, 20.3–23.1 °C) during the entire study. 

### 2.3. Ethical Approval Statement

Animal handling experiments and tests were implemented agreeing with the suitable procedures and regulations of the Ethical Conduct for the Use of Animals in Research at King Faisal University. Additionally, the Institutional Animal Care and Use Committee of King Faisal University permitted the experimental protocol with the ethical approval number KFU-REC-2022-FEB-EA000417. 

### 2.4. Induction Renal Ischemia/Reperfusion (I/R) Injury

Wistar male rats were fasted for 8 h, then anesthetized using isoflurane with oxygen (2%, 0.5 L/h) and placed on a surgical heating pad to maintain the rats’ body temperature at 36–38 °C throughout the whole renal ischemia/reperfusion (I/R) surgery. Under sterilized conditions, a midline cut was performed to distinguish the kidneys situated within the retroperitoneal area. Two non-traumatic vascular clamps were utilized to occult both left and right pedicles bilaterally for 45 min, creating renal ischemia as indicated by the pale kidney color [17,21]. After 45 min, the clamps were removed, and the kidneys were observed for 5 min until the color turned reddish-brown, indicating reperfusion phase manifestation. Surgical sutures were used to close the abdominal wall, including the muscular layer and skin. Twenty-four hours after renal ischemia/reperfusion (I/R) surgery, animals were anesthetized again using the method mentioned above. Blood samples were acquired via cardiac punctures and then centrifuged (20 min, 5000× *g*) to attain serum. Serum samples were stored at −80 °C to be used later for different serum biochemical examinations. For biochemical analyses, the right kidney was rapidly dissected, homogenized, and kept at −80 °C for biochemical studies. The left kidney was preserved in 10% formalin for histopathological and immunohistochemical examination.

### 2.5. Experimental Design

Rats were randomized into five groups (*n*  =  6); in the Sham group, the animals were subjected to bilateral renal artery dissection without renal artery occlusion. In the Sham + geraniol group, the animals were administered geraniol (Cat. No; 163333, Merck (Sigma-Aldrich, St. Louis, MO, USA)) in a dose of 200 mg/kg, dissolved in 1% Carboxymethylcellulose (CMC) in saline orally using gastric gavage for 14 days, and the animals were treated similarly to the sham group. In the Renal I/R group, the animal was subjected to complete renal ischemia/reperfusion (I/R) surgery as indicated above. In Renal I/R + geraniol groups, the rats were administered geraniol 100 mg/kg or 200 mg/kg orally for 14 days before being confronted with renal ischemia/reperfusion (I/R) surgery [22,23], as mentioned above.

### 2.6. Evaluation of Renal Function

Renal function was checked by measuring serum levels of creatinine (Cr, Cat. No; ab700460), uric acid (Cat. No; ab65344), blood urea nitrogen (BUN, Cat. No; ab83362), lactate dehydrogenase (LDH, Cat. No; ab102526) and kidney injury molecule-1 (Kim-1, Cat. No; ab119597), using the indicated colorimetric or ELISA kit acquired from Abcam Inc. (Boston, MA, USA). The procedures were performed in harmony with the manufacturer’s directions using a spectrophotometer (LEICA UNISTAT^®^; Leica Inc., Allendale, NJ, USA). 

### 2.7. Evaluation of Renal Oxidative Stress Status 

Malondialdehyde (MDA, Cat. No; ab238537), glutathione peroxidase (GPx, Cat. No; ab102530), and glutathione content (GSH, Cat. No; ab239727) ELISA kits were procured from Abcam Inc. (Cambridge, UK). Superoxide dismutase (SOD, Cat. No; MBS036924), catalase (CAT, Cat. No; MBS726781), and ELISA kits were purchased from MyBioSource (San Diego, CA, USA) and performed according to the manufacturer’s instructions. 

### 2.8. Molecular Docking Study

A 3D X-ray model of hTLR4/MD2 [24] (PDB ID: 4G8A, resolution: 2.4 Å), hTLR2 [25] (PDB ID: 6ING, resolution: 2.35 Å) and hNrf2/Keap1 complex [26] (PDB ID: 4L7B, resolution: 2.41 Å) were obtained from the protein databank (accessed on 21 March 2022) (http://www.rcsb.org/), and they were prepared for molecular docking in Autodock Vina [27,28], running in Chimera [29] software. The receptors preparation involved the removal of the surplus copies of the protein chains, water molecules, non-bonded inhibitors, and cofactors, adding polar hydrogens and merging non-polar hydrogens, and finally, default Gasteiger charges were assigned to all the atoms. The rotatable bonds in the residues were detected automatically by Chimera, and they were left free. Geraniol was docked in the three proteins using AutoDock Vina 1.1.2 software. Each molecular docking task was performed with the exhaustiveness parameter set to 8, and the top 10 scoring poses for geraniol in each docking process were examined. 

### 2.9. Gene Expression by Real-Time PCR (qPCR) 

Gene expression for the TLR pathway including TLR2, TLR4, MYD88, and NFκB; for the Nrf2 pathway, including Nrf2, HO-1, and NQO-1; and for apoptotic markers, including Bax and Bcl2 were quantified via real-time PCR (qPCR), consuming the primers’ sequences in compliance with the method described elsewhere [30]. Briefly, RNA was isolated and purified using the Trizol reagent kit (Invitrogen, Carlsbad, CA, USA), then reverse transcribed using the reverse transcription-polymerase chain reaction (RT-PCR) kit (TaKaRa, Kusatsu, Shiga, Japan), following the manufacturer’s procedures. qPCR was applied using SYBR ExScript RT-PCR kit, and quantification examinations were completed via Opticon-2 Real-time PCR reactor (MJ Research, Capital Court, Reno, NV, USA). qPCR results were obtained using Step PE Applied Biosystems (Waltham, MA, USA) software. Target gene expressions were evaluated and correlated to the β-actin as the reference gene, and the results are shown in figures as relative expressions. The primer sequences used in this study were mentioned in Table 1.

### 2.10. Evaluation of Nrf2 Pathway Protein Expression 

Western blot was performed according to the method described previously [30]. Briefly, cytoplasmic and nuclear proteins were extracted from the kidney tissue using the Protein Extraction Kit (KeyGen Biotech Co. Ltd., Nanjing, China), containing protease inhibitor and phosphatase inhibitor cocktails (Sigma Aldrich, Darmstadt, Germany), according to the manufacturer’s protocols. The total protein extracted concentration was considered using a NanoDrop Lite spectrophotometer (Thermo Fisher Scientific, Waltham, MA, USA). Afterward, 50 µg of the total extracted protein was separated via sodium dodecyl sulfate (SDS)-polyacrylamide gel electrophoresis (PAGE) and blotted onto PVDF membranes. PVDF membranes were blocked by incubation in Tris-buffered saline (TBS), containing 3% bovine serum albumin and 0.1% Tween 20 for 1 h at room temperature. After washing with TBS containing 0.1% Tween 20, the membranes were incubated firstly with the primary antibodies (1:300 dilution) for 2 h, and then goat antirabbit HRP-conjugated (as secondary antibody; at a 1:5.000 dilution) at room temperature. The chemiluminescence produced from the luminol reagent was detected with the C-DiGit chemiluminescence scanner (LI-COR, Lincoln, NE, USA), and the band intensity was analyzed using the scanner software.

### 2.11. Evaluation of Inflammatory Indicators

To assess geraniol’s anti-inflammatory effect, inflammation markers, including interferon-gamma (IFN-γ), monocyte chemoattractant protein-1 (MCP-1), tumor necrosis factor-alpha (TNF-α), and interleukin 10 (IL-10), were estimated. IFN-γ (Cat. No; ab46107), MCP-1 (Cat. No; ab219045), TNF-α (Cat. No; ab239425), and IL-10 (Cat. No; ab214566) ELISA Kits were attained from Abcam Inc. (Cambridge, UK) and executed following the manufacturers’ instructions. 

### 2.12. Evaluation of Apoptotic Indicators

The action of geraniol administration before renal I/R on the apoptotic markers, such as caspase-3 (ab39401) and caspase 9 (ab65608), were assessed using kits acquired from Abcam Inc. (Cambridge, UK). 

### 2.13. Evaluations Using Histopathological Investigations

Kidney samples were fixed in 10% formalin for 24 h, and then paraffin beeswax tissue blocks were cut to acquire 4 μm thicknesses sections. Renal sections were collected on glass slides, deparaffinized, and stained using Hematoxylin and Eosin (H&E) and Periodic Acid Schiff (PAS) stains to be inspected under a light microscope. Different renal sections were assessed histologically via two blinded pathologists who scored these sections with a specific scale to evaluate the renal tubulointerstitial damages grade. The renal scoring system used was 0 (no damage), 1 (less than 10%), 2 (11–25%), 3 (26–45%), 4 (46–75%), and 5 (more than 76%) as stated before [31]. At least 10 fields (200× magnification) for each renal section were audited and allocated for renal damage.

### 2.14. Statistical Analysis

Data were presented as mean ± SD. For multiple comparisons, one-way ANOVA followed by Tukey–Kramer as a post hoc test was performed. The 0.05 level of probability was used as the significance level. All statistical analyses were performed using Graph Pad software (version 8, San Diego, CA, USA).

## 3. Results

### 3.1. Predictions of Pharmacokinetics, Drug-Likeness, Physicochemical Properties of Geraniol

Pharmacokinetics, drug-likeness, and physicochemical properties of geraniol were estimated using the SwissADME tool [20], Figure 1b. Geraniol was permeable through the blood–brain barrier and exhibited high gastrointestinal absorption. Geraniol fulfilled Lipinski’s rule with no violation, having a molecular weight of 154.25 g/mol (less than 500 g/mol), with one donor and one acceptor’s hydrogen bonds and an octanol-water partition coefficient (MLOGP) value of 2.59 (less than 5) [32]. Furthermore, geraniol showed a topological polar surface area (TPSA) value of 20.23 Å2 (should be between 20 and 130 Å2), only four rotatable bonds, and a Log S value of −2.78 to be classified as soluble [33]. The assessment results identified geraniol to have a satisfactory array of pharmacokinetics, drug-likeness, and physicochemical properties, which ranked the molecule as a potential drug candidate. 

### 3.2. Geraniol Improves Renal Function and Oxidative Stress Markers in Renal I/R Induced Injury 

**Figure 2:**
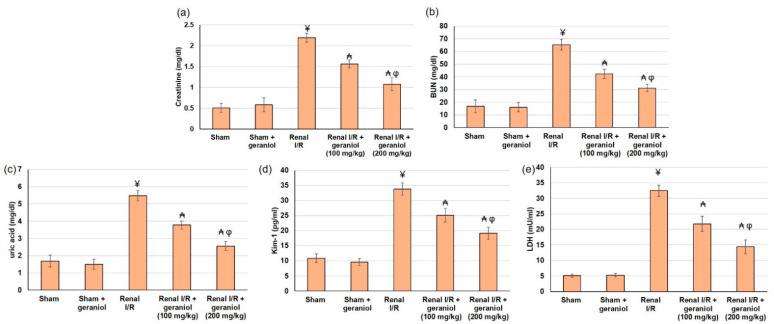
The influences of geraniol (100 and 200 mg/kg) administration for 14 days prior to renal ischemia/perfusion (I/R) induced injury on the renal function assessment, including (**a**) creatinine, (**b**) BUN, (**c**) uric acid, (**d**) Kim-1, and (**e**) LDL. All values are expressed as mean  ±  SD. ¥ indicates statistically significant from the sham group, ₳ indicates statistically significant from the renal I/R group, and φ indicates statistically significant from renal I/R + geraniol 100 mg/kg group (*p* < 0.05) using one-way ANOVA followed by Tukey’s post hoc test.

Renal functions were compromised, as revealed by the significant augmentation in serum Cr, BUN, uric acid, LDL, and Kim-1 levels in renal I/R animals compared to the sham group (*p* < 0.05) (Figure 2). On the other hand, pretreatment with geraniol (100, 200 mg/kg) orally for 14 days prior to renal I/R significantly amended renal functions. Geraniol significantly lowered percentage change, reaching 28.76% and 50.83% for Cr, 35.1% and 52.1 for BUN, 31% and 53.2% for uric acid, 33% and 55.7% for LDL, and 35.62% and 43.37% for Kim-1, compared to renal I/R group.

**Figure 3:**
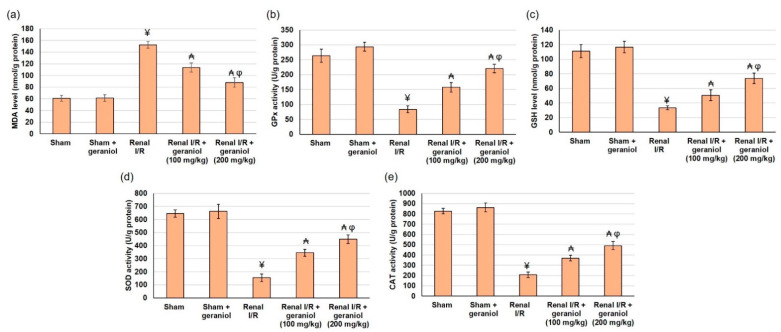
The influences of geraniol (100 and 200 mg/kg) administration for 14 days prior to renal ischemia/perfusion (I/R) induced injury on the antioxidant status, including (**a**) MDA, (**b**) GPx, (**c**) GSH content, (**d**) SOD, and (**e**) CAT. All values are expressed as mean  ±  SD. ¥ indicates statistically significant from the sham group, ₳ indicates statistically significant from the renal I/R group, and φ indicates statistically significant from renal I/R + geraniol 100 mg/kg group (*p* < 0.05) using one-way ANOVA followed by Tukey’s post hoc test.

Additionally, renal I/R animals experienced oxidative stress status as demonstrated by the lowered SOD, CAT, and GPx activities and markedly declined GSH content. Similarly, these animals established elevated lipid peroxidation status as revealed by the amplified MDA level (*p* < 0.05), as shown in Figure 3. Whereas geraniol (100, 200 mg/kg) administered orally for 14 days prior to renal I/R significantly enhanced activities of SOD (345.20 ± 27.13 and 449.18 ± 32.96), CAT (369.70 ± 26.88 and 490.68 ± 39.84), GPx (157.72 ± 16.24 and 221.14 ± 14.19), and GSH content (50.53 ± 7.37 and 73.87 ± 7.10) compared to renal I/R (54.48 ± 29.18 for SOD, 207.45 ± 27.75 for CAT, 83.71 ± 11.66 for GPx, and 33.31 ± 2.58 for GSH content), as shown in Figure 3. These results demonstrated that geraniol restored oxidant/antioxidant equilibrium by reducing lipid peroxidation and increasing GSH level and antioxidant activities in renal tissue compared to the renal I/R group (*p* < 0.05).

### 3.3. Molecular Docking Studies of Geraniol

**Figure 4:**
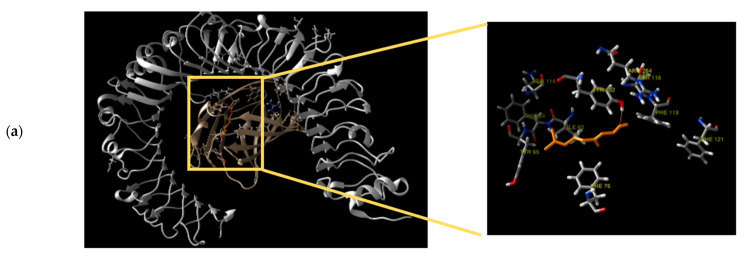

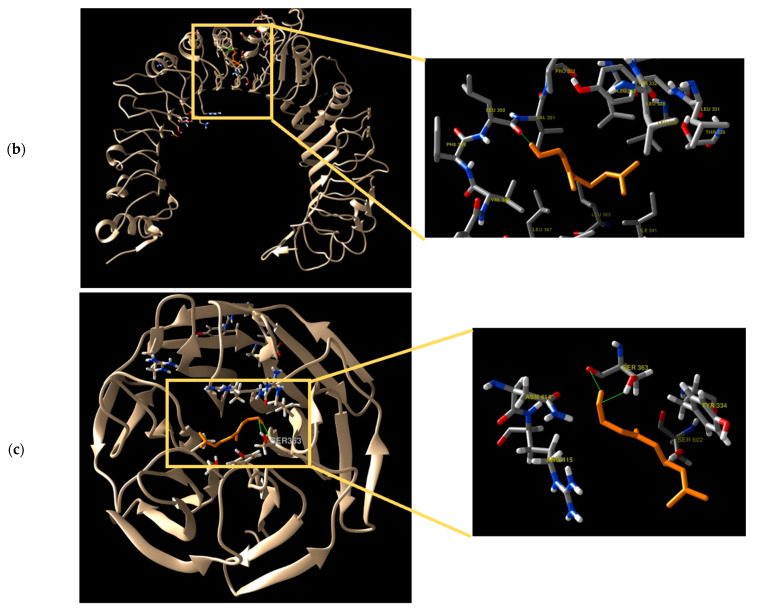
Three-dimensional binding mode of geraniol docking study (**a**) Geraniol (orange) inside the binding site of hTLR4/MD2 (hTLR4 in silver and MD2 in gold), (**b**) Geraniol (orange) inside the binding site of hTLR2, and (**c**) Geraniol (orange) inside the binding site of Keap1 protein.

Geraniol was docked against hTLR4/MD2, hTLR2, and hNrf2/Keap1 protein complexes, and the docking scores and interaction residues of geraniol are shown in Table 2. The docking study revealed that geraniol could fit inside the binding sites of the three different targets through its hydroxy group. Geraniol binds to Tyr102 residue located in the MD2 pocket of hTLR4/MD2 with a 2.3 Å hydrogen bond (Figure 4a), while it binds to Leu350 residue in the case of hTLR2 binding site through a 2.18 Å hydrogen bond (Figure 4b). Geraniol interaction with the Keap1 protein was observed through a hydrogen bond with the residue Ser363 having a length of 1.92 Å (Figure 4c).

### 3.4. Geraniol Prompts Renal Nrf2/HO-1/NQO-1 Pathway in Renal I/R Induced Injury 

**Figure 5:**
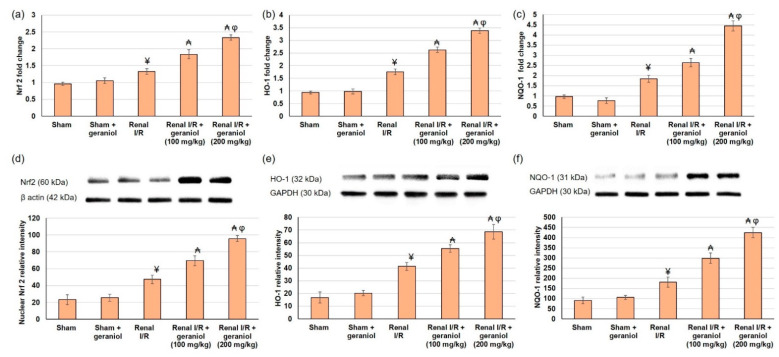
The influences of geraniol (100 and 200 mg/kg) administration for 14 days prior to renal ischemia/perfusion (I/R) induced injury on the renal gene (mRNA) expression of (**a**) Nrf2, (**b**) HO-1, (**c**) NQO-1, and protein expression of (**d**) nuclear Nrf2, (**e**) HO-1, and (**f**) NQO-1. All values are expressed as mean  ±  SD. ¥ indicates statistically significant from the sham group, ₳ indicates statistically significant from the renal I/R group, and φ indicates statistically significant from renal I/R + geraniol 100 mg/kg group (*p* < 0.05) using one-way ANOVA followed by Tukey’s posthoc test.

Cellular responses to oxidative stress induced via renal I/R are partly controlled by the redox-sensitive transcription factors Nrf2, HO-1, and NQO-1. The capability of geraniol to improve renal I/R through the Nrf2 pathway was examined with PCR and western blot analysis. Nrf2, HO-1, and NQO-1 gene and protein expression were slightly escalated in animals that experienced renal I/R, while geraniol further enhanced nuclear Nrf2, HO-1, and NQO-1 gene and protein expressions (Figure 5). Together, these data supported the renal protective actions of geraniol by enhancing Nrf2 nuclear translocation, thus activating the Nrf2/HO-1/ NQO-1 signaling pathway.

### 3.5. Geraniol Averts Renal TLR2,4/MYD88/NFκB Pathway in Renal I/R Induced Injury

**Figure 6:**
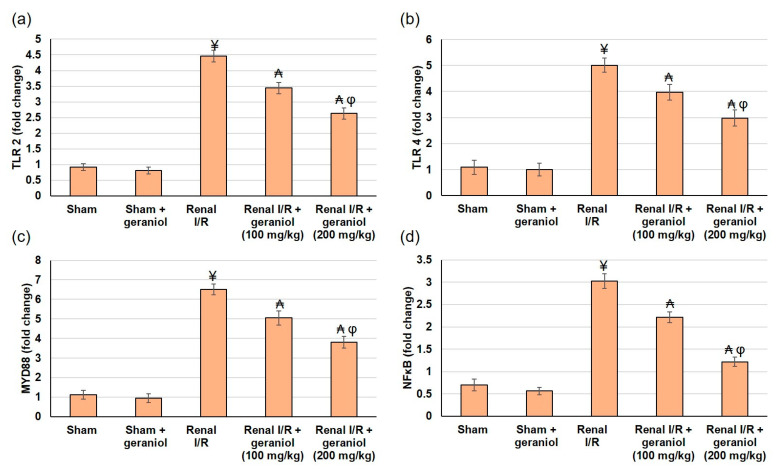
The influences of geraniol (100 and 200 mg/kg) administration for 14 days prior to renal ischemia/perfusion (I/R) induced injury on the renal gene (mRNA) expression of (**a**) TLR2, (**b**) TLR4, (**c**) MYD88, and (**d**) NFκB. All values are expressed as mean  ±  SD. ¥ indicates statistically significant from the sham group, ₳ indicates statistically significant from the renal I/R group, and φ indicates statistically significant from renal I/R + geraniol 100 mg/kg group (*p* < 0.05) using one-way ANOVA followed by Tukey’s post hoc test.

Ischemia/reperfusion-induced stress molecules produced by renal cells trigger TLRs, particularly TLR2 and TLR4, to activate numerous inflammatory mediators, including NFκB [18]. Hence, to inspect the underlying mechanism of geraniol on renal I/R, we assessed the gene expression levels of TLR2, TLR4, and their adaptor protein MYD88, and lastly, NFκB to have an overview of the whole TLR pathway. TLR2, TLR4, and MYD88 were significantly augmented in the I/R group, whereas geraniol administration earlier to I/R significantly (*p* < 0.05) lowered gene expression of TLR2, TLR4, and MYD88, as revealed in Figure 6a–c. As a result of TLR activation, nuclear NFκB p65 was significantly amplified in the I/R animals when related to the sham group. In contrast, the same protein’s level was relatively diminished in animals that received geraniol (Figure 6d).

### 3.6. Geraniol Precludes Renal Inflammatory and Apoptotic Markers in Renal I/R Induced Injury

**Figure 7:**
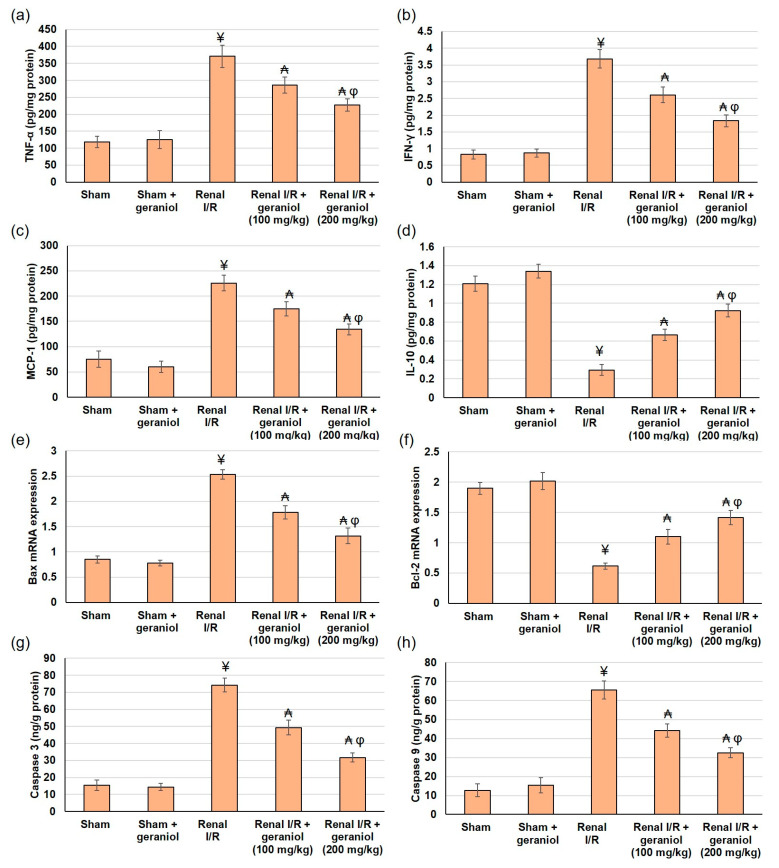
The influences of geraniol (100 and 200 mg/kg) administration for 14 days prior to renal ischemia/perfusion (I/R) induced injury on the renal inflammatory mediators (**a**) TNF-α, (**b**) IFN-γ, (**c**) MCP-1, and (**d**) IL-10, and on apoptosis including gene (mRNA) expression of (**e**) Bax, (**f**) Bcl-2 and (**g**) Caspase 3, and (**h**) Caspase 9. All values are expressed as mean  ±  SD. ¥ indicates statistically significant from the sham group, ₳ indicates statistically significant from the renal I/R group, and φ indicates statistically significant from renal I/R + geraniol 100 mg/kg group (*p* < 0.05) using one-way ANOVA followed by Tukey’s post hoc test.

Inflammation and oxidative stress play a crucial role in the pathogenesis of renal ischemia/reperfusion (I/R) injury [16]. TLR2/4 MyD88-dependent pathway caused NFκB nuclear translocation and activation of downstream signaling resulting in inflammatory and apoptotic reactions. The existing study checked the inflammation response by revealing several cytokines and inflammatory mediators. Renal levels of TNF-α, IFN-γ, and MCP-1 were significantly augmented (*p* < 0.05) in renal I/R animals, while geraniol management prior to kidney ischemia/reperfusion markedly deterred TNF-α, IFN-γ, and MCP-1 boost in I/R rats, as demonstrated in Figure 7a–c, respectively. Conversely the level of IL-10, a cytokine that exerts shielding actions against inflammatory injury, has deteriorated (*p* < 0.05), following renal I/R surgery, whereas geraniol pretreated before renal I/R noticeably increased IL-10 (Figure 7d).

Subsequent to excessive inflammation, oxidative stress, and apoptosis occur; therefore, the current study investigated apoptotic markers Bax, Bcl2, caspase-3, and caspase-9. Following renal I/R, the gene expression levels of Bax, cleaved caspase-3, and caspase-9 activities were significantly amplified, whereas Bcl-2 gene expression level was diminished (Figure 7e–h), suggesting an apoptotic status within the renal I/R animals. However, groups pretreated with geraniol showed ameliorated gene expression levels of Bax, lower activity levels of cleaved caspase-3 and caspase-9, and higher gene expression levels of Bcl-2, signifying that geraniol might limit renal I/R apoptosis (Figure 7e–h). These results suggested that geraniol participated in the regulation of cell apoptosis.

### 3.7. Geraniol Histopathological Effects in Renal I/R Induced Injury

**Figure 8:**
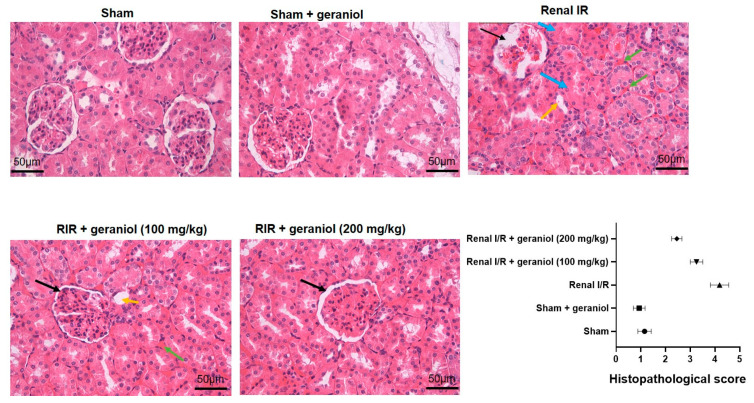
Geraniol’s histopathological effects in renal I/R induced injury. Black arrows show collapsed glomeruli with focal necrosis, blue arrows indicate tubular hypertrophy, green arrows indicate many foci of interstitial hemorrhage, and yellow arrows display sloughed epithelial cells.

The sham group exhibited intact glomeruli and tubules without any unusual pathological changes, as shown in Figure 8. The Renal I/R group displayed interstitial hemorrhage, inflammatory cell infiltration, various degrees of cellular edema and necrosis, and casts within the renal proximal tubules. Whereas the treatment with geraniol noticeably alleviated these pathological fluctuations, as shown in Figure 8. 

## 4. Discussion

Effective drug development processes usually assess the different pharmacokinetic activities of a potential drug candidate, including its ADME. Numerous in silico methods have been increasingly implemented to predict the ADME parameters of potential drugs as an alternative approach to the classical in-lab methods, saving researchers time, money, and efforts. Geraniol, an acyclic monoterpene, exerts many therapeutic and pharmacological activities. The potential of addressing geraniol as a drug candidate was investigated via the SwissADME tool [20], a famous in silico ADME prediction tool. The results reveal geraniol as a probable drug molecule through fulfilling the desired physicochemical properties, drug-likeness features, and desired pharmacokinetics.

Furthermore, the pharmacokinetics of geraniol could impact its drug characteristics and utilization. For example, Geraniol can cross the blood–brain barrier, which is a significant property that could allow the compound to be of some potential in treating brain-related diseases, such as Parkinson’s and Alzheimer’s. Siddique, et al. [34] already showed a protective effect of geraniol on the development of Parkinson’s disease. Additionally, Geraniol displayed a high gastrointestinal absorption, allowing the compound to be administered in oral pharmaceutical dosage forms.

The results from the current study demonstrated that the renal functions were severely compromised, as revealed by the significant augmentation in serum Cr, BUN, uric acid, LDL, and Kim-1 levels in renal I/R animals. Additionally, histological examination showed that the renal I/R group displayed severe histological alterations, indicating renal injury due to renal ischemia-reperfusion (I/R). Previous studies showed that renal I/R increased renal function markers [2,5,16] and several histological alterations [16].

We studied oxidative stress and inflammation with subsequent apoptosis occurring within renal tissue after I/R. Free radicals have a pivotal role in the pathogenesis of renal I/R, facilitating inflammatory cell infiltration, and neutrophil activation [16]. On the other hand, MDA content reflects the content of oxygen free radicals, lipid peroxidation level, and the degree of oxygen free radicals induced damage to the kidney tissue [3]. The transcription factor Nrf2 regulates the expression of antioxidant enzymes, including HO-1, SOD, and NQO-1 [16]. The outcomes of the present study showed renal I/R rats experienced oxidative stress status as demonstrated by the lowered SOD, CAT, and GPx activities and markedly declined GSH content and elevated peroxidation state. Furthermore, Nrf2, HO-1, and NQO-1 gene and protein expression were slightly escalated in animals that experienced renal I/R. Numerous studies indicated that renal ischemia/reperfusion induces nuclear translocation of Nrf2 [7,16], with subsequent augmented levels of HO-1, SOD, and NQO-1 [2,5,16].

Recent work has verified the crucial importance of transmembrane receptors in the injured tubular epithelial cell, most notably TLR, triggered by both exogenous and endogenous ligands in response to external and internal stresses. Indeed, in the current study, TLR2, TLR4, and their adaptor protein MYD88 were significantly augmented in the I/R group. Because of TLR activation, nuclear NFκB p65 was amplified, and TNF-α, IFN-γ, and MCP-1 were significantly augmented in renal ischemia-reperfusion animals. Furthermore, the IL-10 level deteriorated following renal I/R surgery. IL-10 is a cytokine that exerts shielding actions against inflammatory injury. Earlier studies demonstrated that the expression of TLR4 and its downstream factors were significantly amplified, and the nuclear translocation of NF-κB p65 was increased after the renal I/R [16]. Additionally, pro-inflammatory cytokines TNF-α, IL-1β, and IL-6 were significantly increased in IRI rats [2,5,16], and anti-inflammatory cytokine IL-10 was lowered in renal I/R [5].

As a consequence of oxidative stress and inflammation arising within the renal tissue following I/R, apoptotic signaling begins to occur. The up-regulation of NFκB has contributed to the elevated Bax expression and depressed Bcl-2 expression, resulting in an apoptosis [2,7]. We demonstrated that following renal ischemia-reperfusion, the gene expression level of Bax and cleaved caspase-3 and caspase-9 significantly increased, whereas Bcl-2 gene expression level decreased, suggesting an apoptotic status within the renal of I/R animals. Similarly, Fawzy, et al. [2] revealed that renal I/R significantly decreased renal gene levels of Bcl-2, whereas it increased the levels of Bax.

Early and effective regulation of both the oxidative stress as well as the inflammatory response is vital for the prevention and treatment of renal injury [16]. Previously, geraniol relieved different organ injuries; for instance, geraniol alleviated LPS-induced acute lung injury [35], acetaminophen-induced liver injury [22], cisplatin-induced neurotoxicity [13], and others. Our study indicated that geraniol (100, 200 mg/kg) administered orally for 14 days before renal I/R ameliorated renal function, as reflected by the reduced elevation of renal function markers after I/R. A prior study revealed that diabetic rats treated with geraniol exhibited a noticeable improvement in renal function [12]. The mechanisms by which geraniol is protected against renal I/R injury remain vague; therefore, the next step was to study the underlying mechanism responsible for this renoprotection of geraniol. In the current study, the focus was on both the Nrf2 pathway with associated antioxidant activity and TLR2,4/NFκB with subsequent inflammation (after in silico supportive results), and finally the effect of both pathways on apoptosis.

To understand the underlying mechanism, an in silico docking study was established against pharmacologically active target enzymes and proteins. As a hydroxyl compound, geraniol exhibited good fitting properties in the active binding sites of three protein complexes: hTLR4/MD2, hTLR2, and hNrf2/Keap1, with good docking scores ranging from −5.4 to −5.9 kcal/mol. Furthermore, visual inspection of the docking poses showed that geraniol could fit within the main active site through hydrogen bond formation Tyr102, Leu350, and Ser363, in hTLR4/MD2, hTLR2, and hNrf2-Keap1, respectively. These in silico results encouraged further in vivo investigation for Nrf-2/HO-1/NQO-1 and TLR2,4/MYD88/NFκB pathway.

The present study revealed that administration of geraniol before renal I/R significantly enhanced SOD, CAT, GPx, and GSH content, increased GSH levels, and decreased MDA level, demonstrating a relatively mild oxidative injury. These results signified that geraniol restored oxidant/antioxidant equilibrium by reducing lipid peroxidation and antioxidant activities in renal tissue. Numerous earlier studies showed the antioxidant power of geraniol in diabetic rats [12], TNBS-induced colitis [15], liver ischemia-reperfusion [6], 12-O-tetradecanoylphorbol-13-acetate (TPA)-induced oxidative stress and inflammation in mouse skin [36] and 2-acetylaminofluorene induced oxidative stress, inflammation, and apoptosis in the liver [37].

We proposed Nrf2/HO-1/NQO-1 signaling as an antioxidant augmentation mechanism based on an earlier in silico docking study. Nrf2 is a crucial component of cellular redox homeostasis in attenuating oxidative-stress-associated pathological processes [38]. The current investigation indicated that geraniol administration prior to renal I/R geraniol further enhanced nuclear Nrf2, HO-1, and NQO-1 gene and protein expressions. Earlier studies reported that geraniol is a potent activator for the Nrf2 pathway and its downstream target HO-1 in hepatic ischemia-reperfusion injury, which mediated protection against oxidative stress-induced apoptosis in hepatic ischemia-reperfusion injury [6]. Another study showed geraniol-mediated marked protection against atherogenic diet AD-induced abnormalities by the enhancing Nrf2 [38]. Together, these data supported the renoprotective actions of geraniol against oxidative stress via enhancing the Nrf2/HO-1/NQO-1 signaling pathway.

The other mechanism we focused on in the current study is the inflammatory cascade, which is a significant component in the pathogenesis of the renal I/R [22]. Once more, TLRs, especially TLR 2 and 4, were promoted to be potential targets for geraniol based on the former in silico examination. Geraniol (100, 200 mg/kg) administered orally for 14 days prior to renal I/R suggestively lowered expression of TLR2, TLR4, and MYD88 as well as NFκB p65 with subsequent TNF-α, IFN-γ, MCP-1 deterioration, and IL-10 augmentation compared to the ischemic renal group. These results were consistent with other findings reported in the literature indicating the anti-inflammatory activities of geraniol in different models. For instance, treatment with geraniol significantly opposed the TNBS-induced colitis effects on the inflammatory parameters, including NFκB, PGE2, IL-1β, ICAM-1, and MPO [15]. Moreover, Hasan and Sultana [37] demonstrated that pre-treatment with geraniol lowered the expression of caspase-3,9, COX-2, NFκB, PCNA, iNOS, and VEGF; thus, it may be used as a preventive agent against 2-Acetylaminofluorene-induced oxidative stress, inflammation, hyperproliferation, and apoptotic damage in the liver of female Wistar rats. Besides, geraniol pretreatment significantly improved the elevated inflammatory markers TNF-α, iNOS, and COX-2 levels in the hepatic I/R injury group [6]. As for the renal actions, geraniol exhibited protection against cyclosporine A-induced nephrotoxicity via lowering inflammation mediators, including (ICAM-1, IL-18, and NF-κB) [39], protection against methotrexate-induced acute kidney injury via Keap1/Nrf2/HO-1 and MAPK/NF-κB pathways [40]. Geraniol pretreated animals exhibited decreased pro-inflammatory and anti-inflammatory factors, representing that geraniol protected the kidneys against I/R by regulating oxidative stress and inflammatory factors.

In the present study, animals pretreated with geraniol showed ameliorated gene expression levels of Bax and activity levels of cleaved caspase-3 and caspase-9 and higher gene expression levels of Bcl-2, signifying that geraniol might participate in the regulation of cell apoptosis. Earlier, geraniol reduced inflammation and oxidative stress-induced DNA damage, exerting an antiapoptotic effect. Geraniol pretreatment significantly ameliorated this elevation in Bax expression and caspase 3 and 9 levels compared to the hepatic IRI group [6].

Altogether, geraniol’s reno-protection involved anti-inflammatory and antioxidative capabilities as geraniol markedly suppressed the production of proinflammatory factors via inhibition of the TLR4 pathway. They elevated the antioxidative effects via activation of the Nrf2 pathway.

Generally, studying the effect of natural products, especially those isolated from essential oils, on kidney ischemia/reperfusion is not common, and very few studies discuss this approach. One study proved that the monocyclic monoterpene, anethole, had a similar effect to those of geraniol, discovered in our research, in the protection against kidney ischemia/reperfusion [41]. Other essential oils and plant extracts may have similar effects, such as lavender oil [42], Persian sage (*Salvia rhytidia)* [43], *Origanum majorana*, Carvacrol [44], and Nigella sativa [45]. Many other single compounds (natural and synthetic) were studied for their effect on renal ischemia/ reperfusion, however, the majority of these studies were for treatment rather than a protective activity, e.g., propofol [46], Butyrate [47], and others.

## 5. Conclusions

Animals that experienced renal I/R induced injury exhibited compromised renal functions, numerous histological alterations, oxidative stress, inflammation, and apoptotic status. Our in silico and in vivo findings evidenced geraniol to protect against renal I/R status via the inhibition of TLR2,4/MYD88/NFκB pathway (mediate anti-inflammation) and activation of the Nrf2 pathway (mediates antioxidative activities). Geraniol demonstrated an antiapoptotic activity as another mechanism for its antirenal I/R condition. These data might suggest utilizing geraniol pretreatment as a potential new therapeutic approach for protection against renal I/R.

## Figures and Tables

**Table 1:** Primer sequences for all markers used in real-time PCR experiments.

TLR Pathway	Primer Sequence (5′ to 3′)	Gen-Bank Accession Number
Nrf2	5′ CAT TTGTAGATGACCATGAGTCGC 3′ (sense) 5′ ATCAGGGGTGGTGAAGACTG ′ (antisense)	NM_031789.2
HO-1	5′ GTGCACATCGTGCAGAGAA 3′ (sense) 5′ GTGCACATCCGTGCAGAGAA3′ ′ (antisense)	NM_012 580.2
NQO1	5′-AGGATGGGAGGTACTCGATC -3′ (sense) 5′-AGGCGTCCTTCCTTATATGCTA -3′ ′ (antisense)	NM_008706.5
TLR2	5′-ATGAACACTAAGACATACCTGGAG-3′ (sense) 5′-CAAGACAGAAACAGGGTGGAG-3′ (antisense)	NM_198769
TLR4	5′-CATGACATCCCTTATTCAACCAAG-3′ (sense), 5′-GCCATGCCTTGTCTTCAATTG-3′ (antisense)	NM_019178
MyD88	5′-GAGATCCGCGAGTTTGAGAC-3′ (sense) 5′-CTGTTTCTGCTGGTTGCGTA-3′ (antisense)	NM_198130.2
NFκB	5′-ATCATCAACATGAGAAACGATCTGTA-3′ (sense) 5′-CAGCGGTCCAGAAGACTCAG-3′ (antisense)	L26267.1
Bax	5′-GTGGTTGCCCTCTTCTACTTTG-3′ (sense) 5′-CAAAAGATGGTCACTGTCTGC-3′ (antisense)	NM_017059.2
Bcl-2	5′-CCGGGAGATCGTGATGAAGT-3′ (sense) 5′-ATCCCAGCC TCCGTTATCCT-3′ (antisense)	NM_016993.1
β-Actin	5′-TGCTATGTT GCCCTAGACTTCG-3′ (sense) 5′-GTTGGCATAGAG GTCTTTACGG-3′ (antisense)	NM_031144

**Table 2:** Energy score (kcal.mol−1) and interacting residues for binding of geraniol with the three targeted receptors: hTLR4/MD2, hTLR2, and hNrf2/Keap1.

Target Protein	Complex Energy (Kcal/mol)	Interacting Residues
hTLR4/MD2	−5.9	Tyr102
hTLR2	−5.5	Leu350
Keap1	−5.4	Ser363

## Data Availability

Data is contained within the article.
